# Association between glutathione peroxidase‐3 activity and carotid atherosclerosis in patients with type 2 diabetes mellitus

**DOI:** 10.1002/brb3.1773

**Published:** 2020-08-29

**Authors:** Ping Ling, Wanying Shan, Guojie Zhai, Chunfang Qiu, Yuan Liu, Yuan Xu, Xiuyan Yang

**Affiliations:** ^1^ Department of Neurology Suzhou Ninth People's Hospital Suzhou China

**Keywords:** carotid atherosclerosis, carotid plaque, glutathione peroxidase 3, type 2 diabetes mellitus

## Abstract

**Background and Aims:**

Deficiency of glutathione peroxidase 3 (GPx3) has been recognized as an independent risk factor for cardiovascular events. However, little is known regarding the role of GPx3 in carotid atherosclerosis, which is ubiquitously observed in type 2 diabetes mellitus (T2DM). This study aimed to investigate the relationship between GPx3 activity and carotid atherosclerosis among patients with T2DM.

**Methods:**

From January 2018 to December 2018, 245 consecutive patients with T2DM were enrolled in this observational study. Assessment of serum GPx3 activity was performed after admission. We also used carotid ultrasound to measure the mean carotid intima–media thickness (CIMT) and to assess the presence of carotid plaque.

**Results:**

Of the 245 patients, the median serum GPx3 activity was 22.5 U/ml (interquartile range, 12.4–35.9 U/ml). Carotid plaque was observed in 113 (46.1%) patients, and mean CIMT was 0.8 ± 0.1 mm. Univariate analysis showed that age, smoking, previous coronary heart disease, carotid plaque, and level of mean CIMT and hypersensitive C‐reactive protein were significantly associated with decreasing tertile of GPx3. Furthermore, after adjusting for all potential confounders by multivariable logistic regression analysis, PGx3 activity was significantly and independently associated with the mean CIMT (*β* = −.406, *p* = .002) and carotid plaque (first tertile of GPx3, odds ratio, 1.870, 95% confidence intervals, 1.124–3.669, *p* = .024).

**Conclusions:**

This study demonstrated that serum GPx3 activity was inversely associated with mean CIMT and carotid plaque, suggesting that lower GPx3 activity may be an independent predictor for carotid atherosclerosis in T2DM.

## Graphical abstract

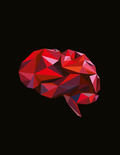

## 1 INTRODUCTION

According to the nationally representative cross‐sectional survey in 2013 in mainland China, the prevalence of diabetes was up to 10.9% (Wang et al., 2017). People with type 2 diabetes mellitus (T2DM) have a higher risk of cardiovascular complications, such as myocardial infarction, ischemic stroke, and carotid atherosclerosis (Henning, 2018; Liu, Baqar, Lincz, & Ekinci, 2019). Ultrasound measurement of carotid intima–media thickness (CIMT) is widely recognized as a non‐invasive and reliable surrogate marker for carotid atherosclerosis (Chen et al., 2016; Everson‐Rose et al., 2014; Nambi et al., 2012; Nezu, Hosomi, Aoki, & Matsumoto, 2016; Plichart et al., 2011). Also, accumulating evidences confirmed a significant association of increasing CIMT level with cardiovascular event risk in T2DM patients (Matsumoto, Sera, Nakamura, Ueki, & Miyake, 2002; Melidonis et al., 2003; Mitsuhash et al., 2002). Therefore, identifying the risk factors and potential mechanisms of carotid atherosclerosis in T2DM subjects is greatly important for early intervention of future vascular events.

Inflammation and oxidative stress are milestones of the atherosclerosis and are supposed to take place in the initiation, progression, and rupture of lipid‐rich lesions (Viña, Borras, Abdelaziz, Garcia‐Valles, & Gomez‐Cabrera, 2013; Yang et al., 2017). Several serological biomarkers of oxidative stress, such as matrix metalloproteinases, lipid hydroperoxides, and NADPH oxidase, have been reported to be associated with atherosclerosis (Tibaut et al., 2019). Glutathione peroxidase‐3 (GPx3), previously known as extracellular glutathione peroxidase, is a selenoprotein antioxidant enzyme that protects the organism from oxidative damage and maintains vascular homeostasis (Lee et al., 2008; Voetsch & Loscalzo, 2004). GPx3 deficiency has been found to be associated with increases of peroxide‐related oxidants, suppression of insulin signaling (Chung et al., 2009), and subsequent chronic diseases such as cardiovascular events (Pastori et al., 2016) and chronic kidney disease (Pang et al., 2018). However, the relationship between GPx3 activity and carotid atherosclerosis has not been well established. Therefore, we performed this hospital‐based observational study in a cohort of patients with T2DM to investigate the relationship between GPx3 activity with CIMT and carotid plaque.

## 2 MATERIALS AND METHODS

### 2.1 Patients

During January 2018 to December 2018, we prospectively recruited patients who hospitalized in Suzhou Ninth People's Hospital for treatment of T2DM. The diagnosis of T2DM was based on World Health Organization criteria (Alberti & Zimmet, 1998). The exclusion criteria were as follows: (a) less than 18 years old; (b) other types of diabetes; (c) unstable medical conditions such as severe pneumonia, malignancy, overt renal dysfunction, and hepatic insufficiency; (d) autoimmune diseases, inflammatory diseases, or other endocrine disorders. Informed consent was obtained from all participants or legal representatives. The study was approved by the Ethics Committee of Suzhou Ninth People's Hospital.

### 2.2 Baseline data collection

Data collection was performed using a standardized questionnaire by trained physician after admission. Variables were recorded as follows: demographic characteristics (age and gender), vascular risk factors (hypertension, hyperlipidemia and smoking), previous medication (antihypertensive, antidiabetic, antiplatelet and statin medication), and clinical data (history of cardiovascular disease, body mass index, systolic blood pressure, and diastolic blood pressure). Body mass index was calculated as the weight in kilograms divided by the square of height in meters. Hypertension was defined as systolic blood pressure level ≥140 mmHg and/or diastolic blood pressure level ≥90 mmHg on three occasions, a self‐reported physician diagnosis of hypertension, or receiving antihypertensive medication. Hyperlipidemia was defined as total cholesterol ≥5.0 mmol/L, low‐density lipoprotein cholesterol ≥3.0 mmol/L, or receiving lipid‐lowering medication (Graham et al., 2007; Schroeder et al., 2003).

### 2.3 Biochemical measurements

Venous blood samples were drawn into standardized tubes containing an anticoagulant within 24 hr after admission. For assessing the GPx3, the specimens were immediately separated by centrifugation at 1,500 r/min for 10 min and the isolated serum frozen at −80°C for subsequent analysis. The measurement of GPx3 activity was conducted by colorimetric assay kit (Abcam) following the manufacturers' instructions. All measurements were performed by duplicate, and inter‐ and intra‐assay coefficients of variation were 6.0% and 4.1%, respectively. The lower functional detection limit of the assay is 0.5 mU/ml. We also performed laboratory tests including fasting blood glucose, hemoglobin A1c (HbA1c), high‐sensitivity C‐reactive protein (hs‐CRP), homocysteine (HCY), and serum lipid status.

### 2.4 Carotid ultrasonography

Carotid ultrasonography on each subject was performed by a high‐resolution and linear‐array transducers (Philips iU22; Philips), with a linear probe at frequency of 4.0–9.0 Hz. The IMT of the common carotid artery, carotid bulb, and internal carotid artery were measured in both arteries. For the common carotid artery, both near and far walls were examined 10 mm proximal to the bulb. Only far walls were examined in bulb and internal carotid artery. Mean CIMT was calculated by averaging the left and right IMT. Plaque was defined as a focal structure that encroaches into the arterial lumen of ≥0.5 mm or 50% of the surrounding IMT value (Ng, Lim, Kang, Kadir, & Tai, 2016; Touboul et al., 2007). All ultrasonographic examination was performed by a single certified sonographer who was blinded to clinical data.

### 2.5 Statistical analysis

The statistical analysis was performed using SPSS version 22.0 (SPSS Inc.). We performed the Kolmogorov–Smirnov test to evaluate the normality of continuous variables. Continuous variables were presented as mean ± standard deviation or median (interquartile range), and categorical data were expressed as *n* (%). Differences in baseline characteristics among the GPx3 tertile were tested using the analysis of variance or Kruskal–Wallis test for continuous variables, and Pearson's chi‐square test for categorical variables. We performed logistic regression models to detect the risk factors for increasing value of mean CIMT and carotid plaque. Multivariable analysis was adjusted for all potential confounders with statistically significant association at *p* < .1 in univariate regression analysis. A value of *p* < .05 was considered statistically significant in all analyses.

## 3 RESULTS

A total of 245 patients with T2DM were included for the final analysis. The mean age was 63.8 ± 8.3 years, and 53.1% of the participants were male. Among these patients, 66.5% had hypertension, 18.8% had hyperlipidemia, and 26.5% were current smokers. After admission, carotid plaque was observed in 113 (46.1%) patients, and mean CIMT was 0.8 ± 0.1 mm.

The median GPx3 value was 22.5 U/ml, with tertile levels as follows: first tertile <18.5 U/ml, second tertile 18.5–32.6 U/ml, and third tertile >32.6 U/ml. The baseline characteristics were compared between the groups set according to serum GPx3 tertile (Table 1). The participants with lower activity of GPx3 was more likely to be older (*p* = .009) and to have higher mean CIMT (*p* = .004) and hs‐CRP (*p* = .012) levels, as well as to have the history of smoking (*p* = .025), previous coronary heart disease (*p* = .042), and carotid plaque (*p* = .049).

**TABLE 1:** Baseline characteristics of the study population according to the glutathione peroxidase‐3 activity tertile

Variables	1st tertile (*n* = 80)	2nd tertile (*n* = 83)	3rd tertile (*n* = 82)	*p* Value
Demographic characteristics				
Age, year	65.9 ± 8.4	63.6 ± 8.2	61.9 ± 7.7	.009
Male (%)	43 (35.8)	44 (53.0)	43 (52.4)	.985
Vascular risk factors				
Hypertension (%)	51 (63.8)	56 (67.5)	56 (68.3)	.809
Hyperlipidemia (%)	15 (18.8)	18 (21.7)	13 (15.9)	.631
Smoking (%)	30 (37.5)	17 (20.5)	18 (22.0)	.025
Previous ischemic stroke (%)	14 (17.5)	9 (10.8)	11 (13.4)	.465
Previous coronary heart disease (%)	15 (18.8)	9 (10.8)	5 (6.1)	.042
Clinical data				
Antihypertensive medication (%)	27 (33.8)	27 (32.5)	32 (39.0)	.651
Antidiabetic medication (%)	60 (75.0)	60 (72.3)	52 (63.4)	.239
Antiplatelet medication (%)	11 (13.8)	19 (22.9)	19 (23.2)	.234
Statin medication (%)	16 (20.0)	14 (16.9)	14 (17.1)	.845
Systolic blood pressure, mmHg	137.2 ± 15.6	139.8 ± 18.9	141.9 ± 16.2	.224
Diastolic blood pressure, mmHg	81.4 ± 10.2	79.4 ± 11.7	83.0 ± 11.5	.127
Body mass index, kg/m^2^	25.3 ± 3.0	24.7 ± 2.9	24.5 ± 2.6	.152
Carotid plaque (%)	43 (53.8)	41 (49.4)	29 (35.4)	.049
Mean CIMT, mm	0.8 ± 0.1	0.8 ± 0.1	0.7 ± 0.1	.004
Laboratory data				
Total cholesterol, mmol/L	3.9 ± 0.9	4.0 ± 0.9	3.9 ± 1.0	.671
Triglyceride, mmol/L	1.5 (1.1, 1.9)	1.4 (1.1, 17)	1.4 (1.0, 1.7)	.225
Low‐density lipoprotein, mmol/L	2.3 (2.0, 2.9)	2.3 (1.7, 2.8)	2.0 (1.7, 2.9)	.263
High‐density lipoprotein, mmol/L	1.0 (0.8, 1.1)	1.0 (0.9, 1.2)	1.0 (0.8, 1.1)	.496
Hypersensitive C‐reactive protein, mg/L	4.6 (2.6, 8.5)	4.3 (2.2, 7.2)	2.7 (1.0, 5.8)	.012
Homocysteine, µmol/L	16.4 ± 5.7	15.4 ± 5.0	15.1 ± 5.9	.293
Fasting blood glucose, mmol/L	7.9 ± 2.2	8.3 ± 2.0	7.7 ± 1.5	.125
Hemoglobin A1c, %	7.6 ± 1.8	7.5 ± 1.5	7.2 ± 1.4	.182

Table 2 showed the results of the linear logistic regression analysis of mean CIMT. In univariate linear regression analysis, the mean CIMT level was significantly associated with age (*β* = .243, *p* = .002), smoking (*β* = .145, *p* = .023), previous ischemic stroke (*β* = .141, *p* = .027), previous coronary heart disease (*β* = .182, *p* = .004), body mass index (*β* = .144, *p* = .024), low‐density lipoprotein (*β* = .207, *p* = .004), hs‐CRP (*β* = .178, *p* = .005), HCY (*β* = .199, *p* = .004), HbA1c (*β* = .173, *p* = .007), and GPx3 activity (*β* = −.455, *p* = .003). Furthermore, after adjusting for all potential confounders (age, smoking, previous ischemic stroke, previous coronary heart disease, and body mass index, low‐density lipoprotein, hs‐CRP, HbA1c and HCY level), lower GPx3 activity was identified as an independent predictor of increased mean CIMT (*β* = −.370, *p* = .002).

**TABLE 2:** Multivariate linear regression analysis for associated factors with mean CIMT

Variables	Univariate logistic regression analysis			Multivariate logistic regression analysis^a^		
	*β*	95% CI	*p* Value	*β*	95% CI	*p* Value
Demographic characteristics						
Age	.243	0.002–0.006	.002	.157	0.001–0.004	.012
Male	.033	−0.024 to 0.041	.610			
Vascular risk factors						
Hypertension	−.046	−0.022 to 0.047	.471			
Hyperlipidemia	−.021	−0.048 to 0.034	.740			
Smoking	.145	0.006–0.078	.023			
Previous ischemic stroke	.141	0.006–0.078	.027			
Previous coronary heart disease	.182	0.023–0.121	.004			
Clinical data						
Antihypertensive medication	.058	−0.018 to 0.049	.369			
Antidiabetic medication	.079	−0.019 to 0.057	.364			
Antiplatelet medication	−.072	−0.063 to 0.017	.259			
Statin medication	.026	−0.034 to 0.051	.688			
Systolic blood pressure	−.014	−0.002 to 0.009	.826			
Diastolic blood pressure	−.036	−0.009 to 0.008	.580			
Body mass index	.144	0.002–0.017	.024	.114	0.002–0.015	.043
Laboratory data						
Total cholesterol	.018	−0.015 to 0.024	.783			
Triglyceride	−.002	−0.023 to 0.022	.981			
Low‐density lipoprotein	.207	0.014–0.057	.004	.150	0.005–0.406	.014
High‐density lipoprotein	.047	−0.014 to 0.031	.468			
Hypersensitive C‐reactive protein	.178	0.003–0.011	.005			
Homocysteine	.199	0.002–0.007	.004	.176	0.001–0.008	.004
Fasting blood glucose	−.067	−0.013 to 0.004	.297			
Hemoglobin A1c	.173	0.004–0.026	.007	.126	0.002–0.018	.047
Glutathione peroxidase‐3	−.455	−0.006 to −0.003	.003	−.370	−0.005 to −0.003	.002

^a^ Multivariable analysis was adjusted for age, smoking, previous ischemic stroke, previous coronary heart disease, body mass index, low‐density lipoprotein, hypersensitive C‐reactive protein, homocysteine, and hemoglobin A1c levels.

Table 3 demonstrated the results of the binary logistic regression of the carotid plaque. After adjusting for all potential confounders (age, hypertension, smoking status, previous ischemic stroke, previous stain therapy, and level of triglyceride, HCY, and HbA1c), GPx3 activity in the first tertile (third tertile used as the reference value) was identified as an independent risk factor of carotid plaque (odds ratio, 1.870, 95% confidence intervals, 1.124–3.669, *p* = .024).

**TABLE 3:** Multivariate logistic regression analysis for associated factors with carotid plaque T2MD

Variables	Univariate logistic regression analysis			Multivariate logistic regression analysis^a^		
	OR	95% CI	*p* Value	OR	95% CI	*p* Value
Demographic characteristics						
Age	1.035	1.003–1.065	.022			
Male	0.879	0.531–1.454	.615			
Vascular risk factors						
Hypertension	1.943	1.124–3.358	.017	1.732	1.049–3.161	.044
Hyperlipidemia	0.977	0.531–1.859	.943			
Smoking	3.071	1.694–5.567	.001	2.568	1.360–4.849	.005
Previous ischemic stroke	1.577	1.760–3.269	.021			
Previous coronary heart disease	1.103	0.508–2.397	.604			
Clinical data						
Antihypertensive medication	1.183	0.699–2.002	.531			
Antidiabetic medication	1.140	0.657–1.978	.640			
Antiplatelet medication	1.042	0.556–1.951	.898			
Statin medication	1.893	0.976–3.671	.069			
Systolic blood pressure	1.066	0.992–1.022	.393			
Diastolic blood pressure	1.003	0.981–1.026	.774			
Body mass index	1.065	0.975–1.164	.161			
Laboratory data						
Total cholesterol	1.050	0.797–1.383	.730			
Triglyceride	1.328	1.008–2.063	.048			
Low‐density lipoprotein	1.257	0.896–1.764	.185			
High‐density lipoprotein	0.710	0.373–1.349	.295			
Hypersensitive C‐reactive protein	1.018	0.956–1.085	.573			
Homocysteine	1.080	1.028–1.136	.002	1.061	1.006–1.118	.028
Fasting blood glucose	1.045	0.918–1.190	.504			
Hemoglobin A1c	1.141	1.070–1.343	.041	1.096	1.014–1.313	.043
Glutathione peroxidase‐3						
1st tertile	2.124	1.130–3.992	.019	1.870	(1.124–3.669)	.024
2nd tertile	1.784	0.995–3.332	.069	1.615	0.802–3.252	.179
3rd tertile	Reference	Reference		Reference	Reference	

^a^ Multivariable analysis was adjusted for age, hypertension, smoking, previous ischemic stroke, statin medication, triglyceride, homocysteine, and hemoglobin A1c levels.

## 4 DISCUSSION

This hospital‐based observational study illustrated that lower serum GPx3 activity at admission was independently and significantly correlated to mean CIMT and presence of carotid plaque in a cohort of T2DM patients. Furthermore, these associations remained significant even after adjusting for potential confounders, indicating that decreased GPx3 activity may play a crucial role in carotid atherosclerosis.

It is well‐accepted that atherosclerosis is a pathophysiological process involving of chronic inflammation and oxidative stress, which may cause the vascular endothelium and lead to the cardiovascular diseases. GPx functions as the major antioxidant molecule in the peripheral blood. It may also exert an important anti‐atherogenic effect. To date, several studies have analyzed the GPx activity in human subjects and its prognostic value in atherosclerotic diseases (Buijsse et al., 2017; Lapenna et al., 1998; Pastori et al., 2016). In carotid atherosclerotic plaques, GPx1 activity is found decreased or absent, which may further induce the development of more severe lesions in humans (Lapenna et al., 1998). Moreover, decreased serum GPx3 activity is associated with increased cardiovascular mortality in individuals with low HDL (Buijsse et al., 2017), suggesting that a low antioxidant status predisposing to poor vascular outcomes. Recently, the serum GPx3 activity has been measured in participants with atrial fibrillation and the results showed that decreasing GPx3 activity may be a predictor of fatal and nonfatal cardiovascular complications (Pastori et al., 2016). However, little is known about the role of GPx3 in carotid atherosclerosis. Our present study indicates that the reduced GPx3 activity was involved in the deterioration of mean CIMT and the presence of carotid plaque, in support of the linking between GPx3 activity and the pathogenesis of carotid atherosclerosis in T2DM subjects. According to previous studies, it has been suggested that GPx3 is increased in subjects with insulin resistance, overweight or obese, and metabolic syndrome (Baez‐Duarte et al., 2012, 2014). However, we did not find any cross‐sectional association of GPx3 activity with fasting blood glucose (Spearman's correlation analysis, *r* = .018, *p* = .778) and body mass index (Spearman's correlation analysis, *r* = −.089, *p* = .281). These discrepancies might be explained at least in part that the expression of GPx3 is induced by different disease stage (Chung et al., 2009).

Although the underlying mechanism of the regulation of GPx3 activity and its physiological and pathophysiological roles in carotid atherosclerosis remains limited, several speculations may explain this association. Experimental evidences showed that GPx‐3 can catabolize hydrogen peroxide under circumstances of normal metabolism or oxidative insult, therefore preserving the vascular endothelium‐derived vasorelaxation and anti‐atherosclerosis (Takebe et al., 2002). Nonetheless, the prolonged oxidative stress keeps consuming antioxidants, leading to oxidants augment and endothelial dysfunction (Redón et al., 2003). Moreover, in GPx3‐deficient mice models, vascular endothelial damage is also observed (Wolin, 2011). Thus, GPx3 may be a determinant mediator in diseases which may impair endothelial function and result in atherosclerosis. Furthermore, GPx3 plays a role in inflammatory responses. Decreased GPx3 activity has been observed in patients with systemic inflammatory response syndrome and possessed a predictive value for this syndrome (Manzanares et al., 2009). As reported, pro‐inflammatory cytokines can regulate GPx3 production (Agnani et al., 2011; Montague et al., 1998). We therefore assume that low GPx3 activity may reflect an activation status of inflammation, which might cause endothelial dysfunction and arterial stiffness, and contribute to atherosclerosis (Della Corte et al., 2016). However, our data demonstrated that controlling for inflammatory marker such as hs‐CRP only marginally changed the negative relationship between GPx3 and mean CIMT. This observation suggested that measurement of GPx3 activity provides additional information on risk and might be useful in identifying T2DM patients who would benefit from preventive antioxidative treatment.

The present study has several limitations. Firstly, the observational design makes it difficult to establish a causal relationship between GPx3 and carotid atherosclerosis. Secondly, the study was performed in a single‐center with a relatively small sample of subjects. This selection bias would probably generate sampling bias and reduce the power of study. Thirdly, GPx3 activity was measured only once after admission, which may lead to some misclassification of exposure. Finally, other factors associated with oxidative stress, such as arterial stiffness, matrix metalloproteinases, NADPH oxidase, and superoxide dismutase, were not assessed in this study. As was mentioned above, our study should be considered preliminary.

In conclusion, our study suggested that decreasing serum GPx3 activity is associated with the severity of carotid atherosclerosis in T2DM patients. Further large‐sample prospective studies are needed to confirm the causal relationship and evaluate the underlying mechanism between serum GPx3 and carotid atherosclerosis.

## CONFLICT OF INTEREST

The authors report no conflict of interest.

## AUTHOR CONTRIBUTIONS

PL designed the study, conducted statistical analysis, and wrote the draft of the manuscript. WS and GZ conducted statistical analysis and acquired data. CQ and YL acquired data. YX and XY conceived and designed the study, conducted statistical analysis, and supervised this study.

## Data Availability

The data that support the findings of this study are available on request from the corresponding author.
